# Interdisciplinary Management of Bilateral Maxillary Facial Exostoses Associated With Esthetic and Psychosocial Concerns Using Osteoplasty and Gingivoplasty: A Case Report

**DOI:** 10.7759/cureus.114437

**Published:** 2026-08-12

**Authors:** Keily J Portillo Avila, Norby Rodriguez, Nayibe Arenas Perez, Diosbeny N Camacho Grullon, Lina R Tello Orozco

**Affiliations:** 1 Periodontics, Dental Loft, San Pedro Sula, HND; 2 Dentistry, Prisma Odontologia Estetica, Cali, COL; 3 Dentistry, On the Go Braces, West Palm Beach, USA; 4 Dentistry, Expert Dental N.Y.C., New York, USA; 5 Dentistry, Naples Cosmetics Dentist, Naples, USA

**Keywords:** alveolar bone, buccal exostosis, case report, esthetic dentistry, gingivoplasty, maxillary exostosis, osteoplasty, periodontal surgery, psychosocial distress, smile esthetics

## Abstract

Maxillary facial exostoses are uncommon benign bony enlargements that are typically asymptomatic but may become clinically significant when they compromise facial esthetics or negatively affect a patient's psychological well-being. Surgical intervention is generally reserved for patients with functional limitations or esthetic concerns. This report describes the interdisciplinary management of bilateral maxillary facial exostoses in a young adult using psychological assessment, osteoplasty, and gingivoplasty to restore harmonious maxillary contours and improve patient confidence.

A 26-year-old healthy female (American Society of Anesthesiologists (ASA) Physical Status Classification I) presented with bilateral facial maxillary exostoses that produced a prominent anterior maxillary contour. The patient reported significant esthetic dissatisfaction and psychosocial distress that adversely affected her confidence while performing her professional responsibilities as a school counselor. After comprehensive clinical evaluation and psychological assessment confirming appropriate expectations for treatment, the patient elected surgical management rather than prolonged orthodontic therapy. A full-thickness mucoperiosteal flap was elevated, followed by conservative osteoplasty to recontour the excessive facial alveolar bone and gingivoplasty to establish physiologic gingival architecture. The surgical site was closed with 4-0 nylon sutures. Postoperative healing was uneventful, with suture removal at 17 days demonstrating favorable soft tissue healing and substantial reduction of the bony prominence. At six weeks, complete healing and marked improvement in facial esthetics were observed. The patient reported a significant increase in self-confidence and satisfaction with the treatment outcome.

This case demonstrates that interdisciplinary management combining psychological evaluation with conservative periodontal surgery can successfully address esthetic and psychosocial concerns associated with bilateral maxillary facial exostoses. Osteoplasty combined with gingivoplasty represents a predictable treatment option for carefully selected patients seeking immediate esthetic improvement while avoiding prolonged orthodontic treatment.

## Introduction

Maxillary facial exostoses are benign, localized bony protuberances that develop on the cortical surface of the alveolar bone. Although their exact etiology remains uncertain, proposed contributing factors include genetic predisposition, occlusal loading, functional stress, and adaptive bone remodeling. These osseous enlargements are generally asymptomatic and are often discovered during routine dental examinations. Consequently, treatment is not routinely indicated unless they interfere with oral function, prosthetic rehabilitation, oral hygiene, or facial esthetics [1-8].

When facial exostoses occur in the anterior maxilla, they may alter the natural contour of the alveolar process and produce visible prominence of the upper lip and gingival tissues during smiling or speaking. Although these lesions are benign, the associated esthetic deformity may significantly affect a patient's self-image, confidence, and social interactions. For some individuals, the psychological impact of the deformity may become the primary reason for seeking treatment despite the absence of pain or functional impairment [9-13]. Addressing dentofacial esthetic concerns through targeted dental interventions plays a significant role in enhancing patient self-esteem and psychosocial well-being.

Management of maxillary facial exostoses depends on the patient's symptoms, esthetic concerns, and treatment goals. Conservative observation is appropriate for asymptomatic lesions; however, surgical intervention may be indicated when esthetic or functional problems are present. Osteoplasty allows controlled recontouring of excessive alveolar bone while preserving surrounding anatomical structures, and gingivoplasty may be performed simultaneously to establish harmonious gingival architecture and optimize the final esthetic outcome [14-18].

Successful management of esthetically compromising facial exostoses requires careful patient selection and comprehensive treatment planning. When psychosocial concerns are a major component of the chief complaint, evaluation of patient expectations and emotional well-being may contribute to appropriate clinical decision-making and improve overall treatment satisfaction. An interdisciplinary approach can therefore help ensure that surgical intervention addresses both the physical deformity and the patient's esthetic expectations [19].

Despite the benign nature of maxillary facial exostoses, limited attention has been given to cases in which esthetic and psychosocial concerns constitute the primary indication for surgical treatment. This report describes the interdisciplinary management of bilateral maxillary facial exostoses associated with significant esthetic and psychosocial concerns in a young adult using psychological assessment, osteoplasty, and gingivoplasty. The treatment resulted in predictable healing, improved maxillary contours, and a substantial enhancement in the patient's self-confidence and satisfaction with her smile.

## Case presentation

A 26-year-old female patient (American Society of Anesthesiologists (ASA) Physical Status Classification I) [19], with no significant medical history or known drug allergies, presented to the Periodontics Department at Dent Studio LCB, La Ceiba, Honduras, with a chief complaint of prominent bilateral bony enlargements affecting the anterior maxillary facial alveolar process. The patient reported that the deformity had progressively become a significant esthetic concern and negatively affected her self-confidence during social and professional interactions. She worked as a school counselor and explained that the appearance of her smile caused considerable frustration while speaking with students and colleagues, resulting in psychosocial distress despite the absence of pain or functional impairment.

**Figure 1:**
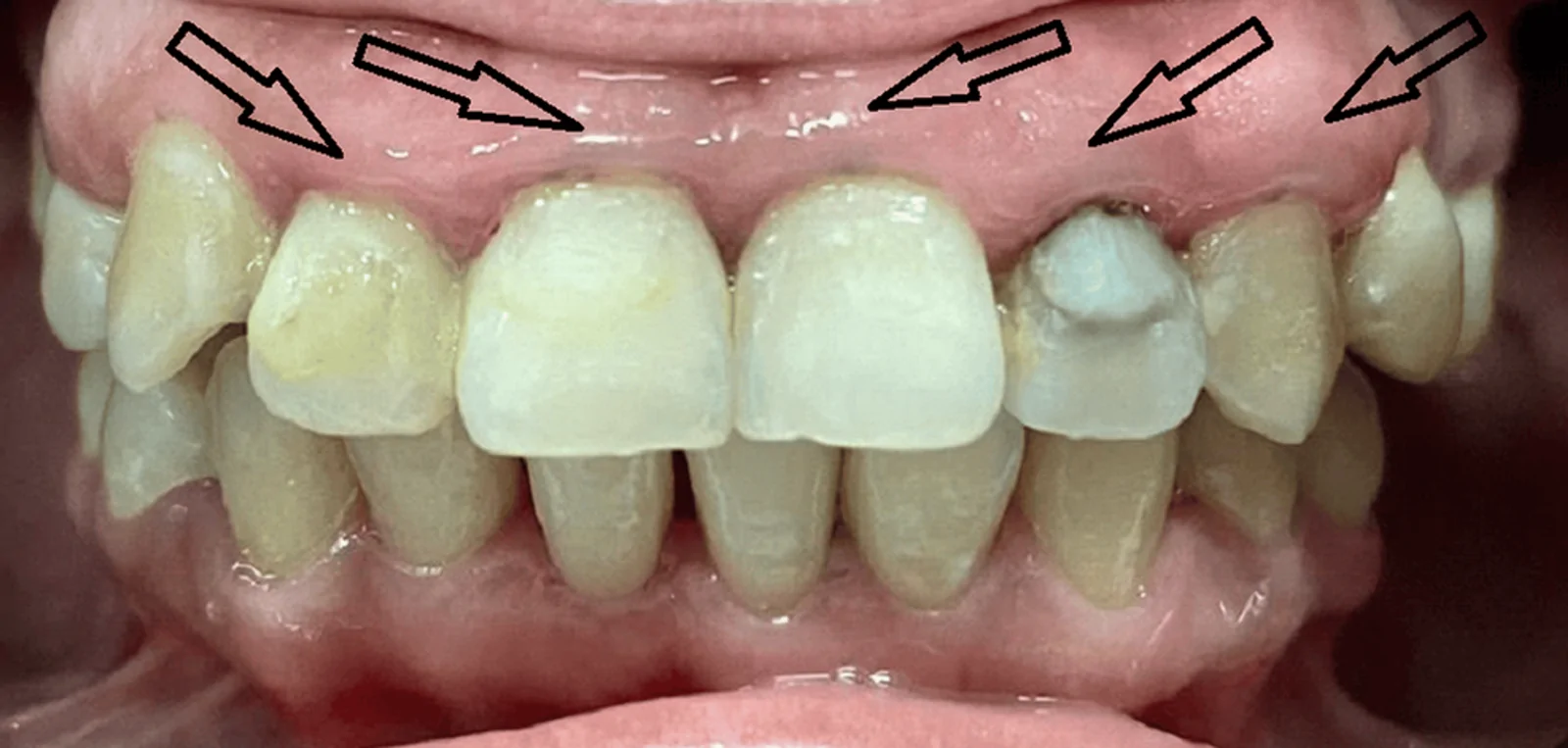
Preoperative clinical appearance of bilateral maxillary facial exostoses. Preoperative intraoral clinical photograph demonstrating prominent bilateral maxillary facial exostoses (black arrows) involving the anterior facial aspect of the maxillary alveolar process. The lesions produced a marked convexity of the facial alveolar contour, which represented the patient's primary esthetic concern and contributed to significant psychosocial distress.

Clinical examination revealed prominent bilateral facial maxillary exostoses extending across the anterior facial aspect of the maxillary alveolar process, producing a marked convexity of the facial alveolar contour. The overlying gingival tissues appeared healthy, with no clinical signs of inflammation or infection (Figure 1).

The patient was informed that the lesions represented benign maxillary facial exostoses and that treatment was elective because the primary indication was esthetic improvement.

Comprehensive orthodontic treatment was discussed as a potential option to improve the overall esthetic appearance. However, the patient declined prolonged orthodontic therapy because of the anticipated treatment duration and expressed a preference for a definitive surgical approach. Given that the patient's chief complaint was primarily related to esthetic dissatisfaction and psychosocial distress, she was referred for psychological evaluation before treatment. The psychologist concluded that the patient demonstrated realistic expectations, understood the benefits and limitations of the proposed procedure, and was an appropriate candidate for surgical intervention. Following psychological clearance, written informed consent for treatment and publication was obtained.

**Figure 2:**
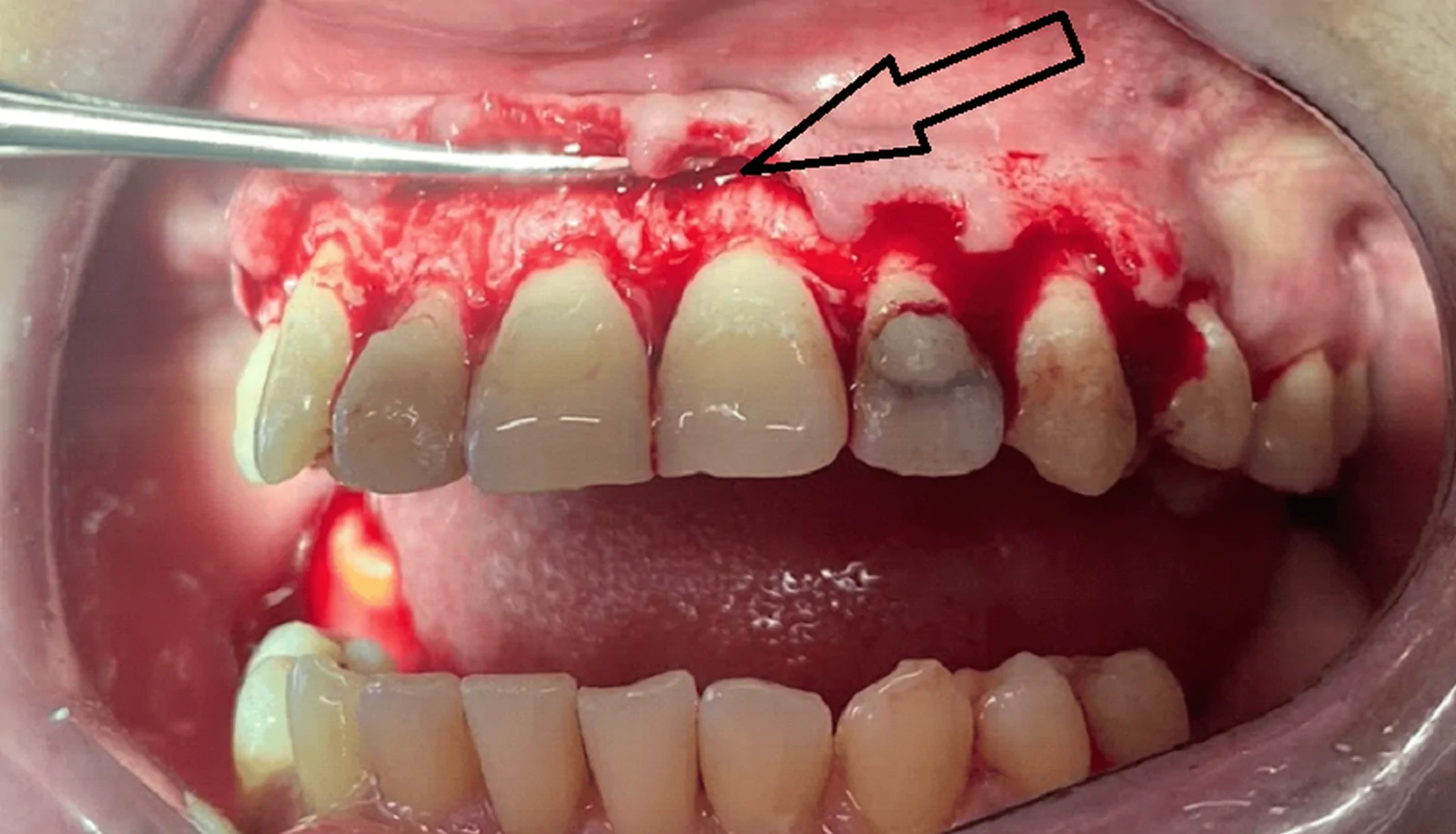
Full-thickness flap elevation and exposure of the maxillary facial exostoses. Intraoperative clinical photograph following elevation of a full-thickness mucoperiosteal flap, exposing the prominent bilateral maxillary facial exostoses (black arrow). The excessive cortical bone was identified before conservative osteoplasty to recontour the facial alveolar process. Gingivoplasty was subsequently performed to establish a harmonious gingival architecture and improve the esthetic outcome.

Under local anesthesia with 2% lidocaine containing 1:100,000 epinephrine, sulcular and crestal incisions were made using a No. 15 scalpel blade. A full-thickness mucoperiosteal flap was carefully elevated to expose the underlying facial alveolar bone (Figure 2).

**Figure 3:**
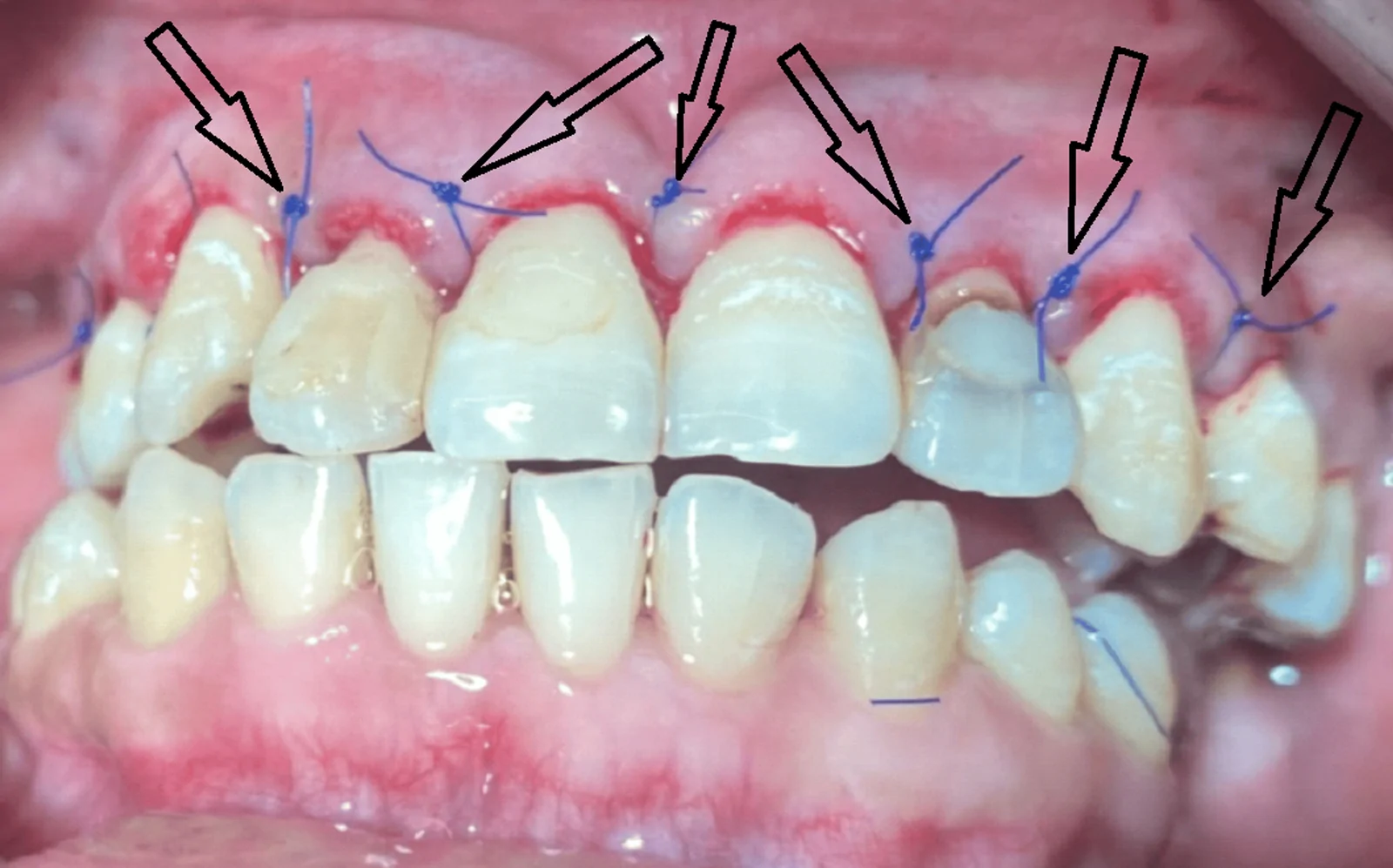
Flap closure following osteoplasty and gingivoplasty. Clinical photograph demonstrating primary flap closure after surgical recontouring of the bilateral maxillary facial exostoses. Following osteoplasty and gingivoplasty, the flap was repositioned and secured with interrupted 4-0 nylon sutures (black arrows), which remained in place for 17 days to facilitate uneventful healing.

Conservative osteoplasty was performed to recontour the excessive cortical bone and restore a more physiologic maxillary contour while preserving the surrounding anatomical structures. Following osseous recontouring, gingivoplasty was completed to establish harmonious gingival architecture and optimize the esthetic outcome. The surgical site was irrigated with sterile saline and closed with interrupted 4-0 nylon sutures (Figure 3).

Postoperative medications included amoxicillin 500 mg orally three times daily for seven days, ibuprofen 600 mg orally three times daily for five days, and a 0.12% chlorhexidine mouth rinse once daily at bedtime for seven days. Written postoperative instructions regarding oral hygiene, dietary recommendations, activity restrictions, and scheduled follow up visits were provided.

**Figure 4:**
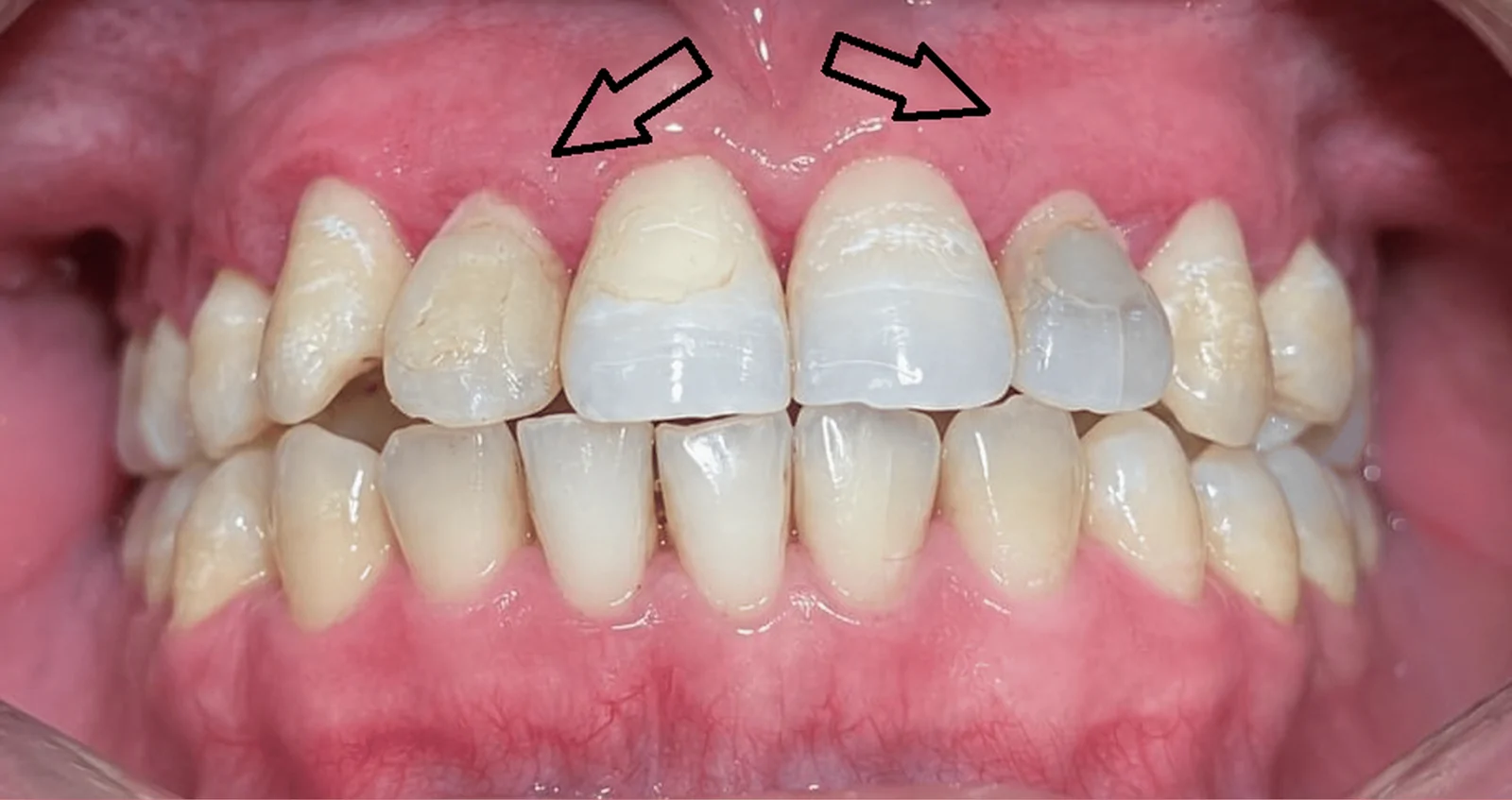
Four-month postoperative clinical outcome following osteoplasty and gingivoplasty. Clinical photograph obtained four months after surgery demonstrating complete soft tissue healing and a harmonious maxillary alveolar contour following conservative osteoplasty and gingivoplasty. The facial alveolar process exhibits a smooth, physiologic contour (black arrows), resulting in an improved esthetic appearance and favorable anatomical architecture with no evidence of recurrence.

Healing was uneventful throughout the postoperative period, with no evidence of infection, wound dehiscence, or other complications. The 4-0 nylon sutures were removed 17 days after surgery, revealing excellent soft tissue healing and a marked reduction in the facial bony prominence. At the six week follow up, complete soft tissue healing and marked improvement in facial esthetics were observed. At the four month follow up, complete soft tissue maturation and restoration of a harmonious maxillary alveolar contour were confirmed, with no evidence of recurrence (Figure 4).

The patient reported complete satisfaction with the esthetic outcome and described a substantial improvement in self-confidence and comfort during professional and social interactions. The final result met the patient's esthetic expectations without the need for prolonged orthodontic treatment.

## Discussion

Facial maxillary exostoses are benign, localized bony enlargements of the cortical alveolar bone that are generally discovered incidentally during routine dental examinations. Their etiology remains incompletely understood, although genetic predisposition, functional loading, parafunctional habits, and adaptive bone remodeling have all been proposed as contributing factors. In most patients, these lesions remain asymptomatic and require no treatment. Surgical intervention is typically reserved for cases in which the exostoses interfere with oral hygiene, prosthetic rehabilitation, speech, mastication, or facial esthetics [1-8].

The present case is notable because the indication for treatment was primarily esthetic rather than functional. Although the patient reported no pain or impairment of oral function, the prominent bilateral facial exostoses produced a visible alteration of the anterior maxillary contour that significantly affected her self-confidence. The psychological impact extended into her professional life as a school counselor, where frequent face-to-face communication made her increasingly self-conscious about her appearance. This case highlights that benign oral conditions may have substantial psychosocial consequences even when they do not produce physical symptoms [9-12].

Before proceeding with surgery, the patient underwent psychological evaluation to confirm that her expectations were realistic and that the perceived esthetic concern was appropriate for surgical management. This interdisciplinary approach helped ensure that treatment addressed both the anatomical deformity and the patient's emotional well-being. Careful assessment of patient expectations is an important component of esthetic treatment planning because postoperative satisfaction depends not only on technical success but also on whether the final outcome aligns with the patient's goals [13-15].

Conservative osteoplasty remains the preferred surgical technique for correcting localized facial exostoses because it permits controlled recontouring of excessive cortical bone while preserving surrounding anatomical structures. In the present case, osseous reduction was combined with gingivoplasty to establish harmonious soft tissue contours and optimize the overall esthetic outcome. This combined approach improved both the underlying bony architecture and the gingival profile, producing a more natural appearance after healing [16-18].

Postoperative healing was uneventful, with excellent soft tissue maturation and no evidence of infection, wound dehiscence, or other complications. By the 17-day follow-up, substantial improvement in the facial contour was already evident, and complete healing at six weeks demonstrated predictable tissue remodeling and satisfactory esthetic integration. The patient's reported improvement in confidence and satisfaction further supported the success of treatment [19].

Reports describing the management of facial maxillary exostoses for primarily esthetic indications remain relatively uncommon in the dental literature. Most published reports emphasize diagnosis or treatment related to prosthetic rehabilitation, whereas fewer describe the psychosocial impact of these lesions or the benefits of combining psychological assessment with periodontal surgical management. Consequently, this case contributes additional clinical evidence supporting an individualized, patient-centered approach when esthetic concerns become the primary indication for treatment.

Although this report describes the successful outcome of a single patient, its findings should be interpreted within the limitations inherent to case reports. Long-term follow-up involving larger numbers of patients would be valuable to further evaluate the stability of surgical outcomes, patient satisfaction, and the recurrence rate following conservative osteoplasty.

Overall, this case demonstrates that interdisciplinary management combining psychological evaluation with conservative periodontal surgery can effectively treat bilateral maxillary facial exostoses when esthetic concerns substantially affect a patient's quality of life. Appropriate patient selection, realistic expectations, meticulous surgical technique, and careful postoperative follow-up are fundamental to achieving predictable functional and esthetic outcomes.

## Conclusions

Conservative surgical management using osteoplasty and gingivoplasty provided a predictable and effective treatment for bilateral maxillary facial exostoses associated with esthetic and psychosocial concerns. In this case, the interdisciplinary approach, including psychological assessment before surgery, facilitated appropriate patient selection and realistic treatment expectations. The procedure resulted in favorable healing, restoration of harmonious maxillary contours, and a substantial improvement in the patient's self-confidence and quality of life. This case highlights the importance of individualized treatment planning and demonstrates that surgical correction may be an appropriate option for carefully selected patients whose benign facial exostoses produce significant esthetic and psychological distress despite the absence of functional symptoms.
